# Supplementary material for: Anion-Responsive Fluorescent Supramolecular Gels
Source: Molecules. 2022 Feb 13;27(4):1257. doi: 10.3390/molecules27041257 (PMC8876235; doi:10.3390/molecules27041257)
Supplement: Supplementary file 1 [file molecules-27-01257-s001.zip › molecules-1587629-supplementary.pdf]

# Anion-Responsive Fluorescent Supramolecular Gels

Giacomo Picci, Matthew T. Mulvee, Claudia Caltagirone, Vito Lippolis, Antonio Frontera, Rosa M. Gomila, Jonathan W. Steed

Index:

**Synthesis and Characterisation** **pag. 2**

**Gels Characterisation** **pag. 9**

**$^{1}$H-NMR Titrations** **pag. 12**

**DFT Calculations** **pag. 15**

**UV–Vis and Fluorescence Studies** **pag. 22**

**Synthesis and Characterisation**

**Scheme S1**. Synthetic pathway followed for the synthesis of **L1**-**L3.**

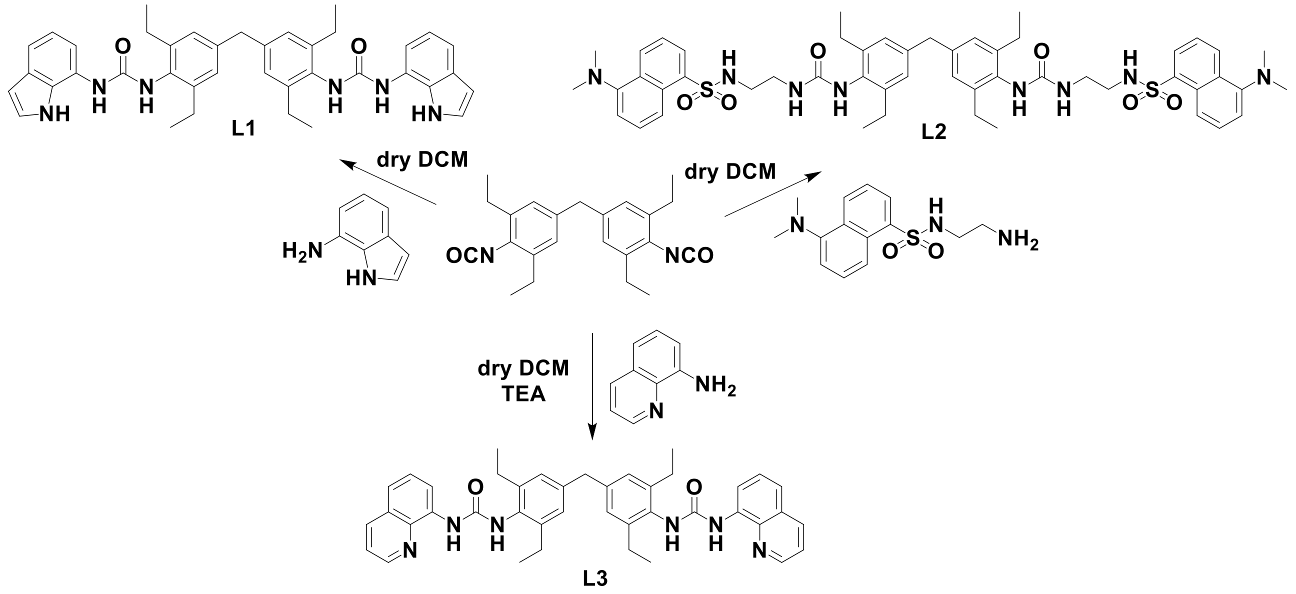


**Scheme S1.** Synthetic pathway followed for the synthesis of **L1**-**L3**.

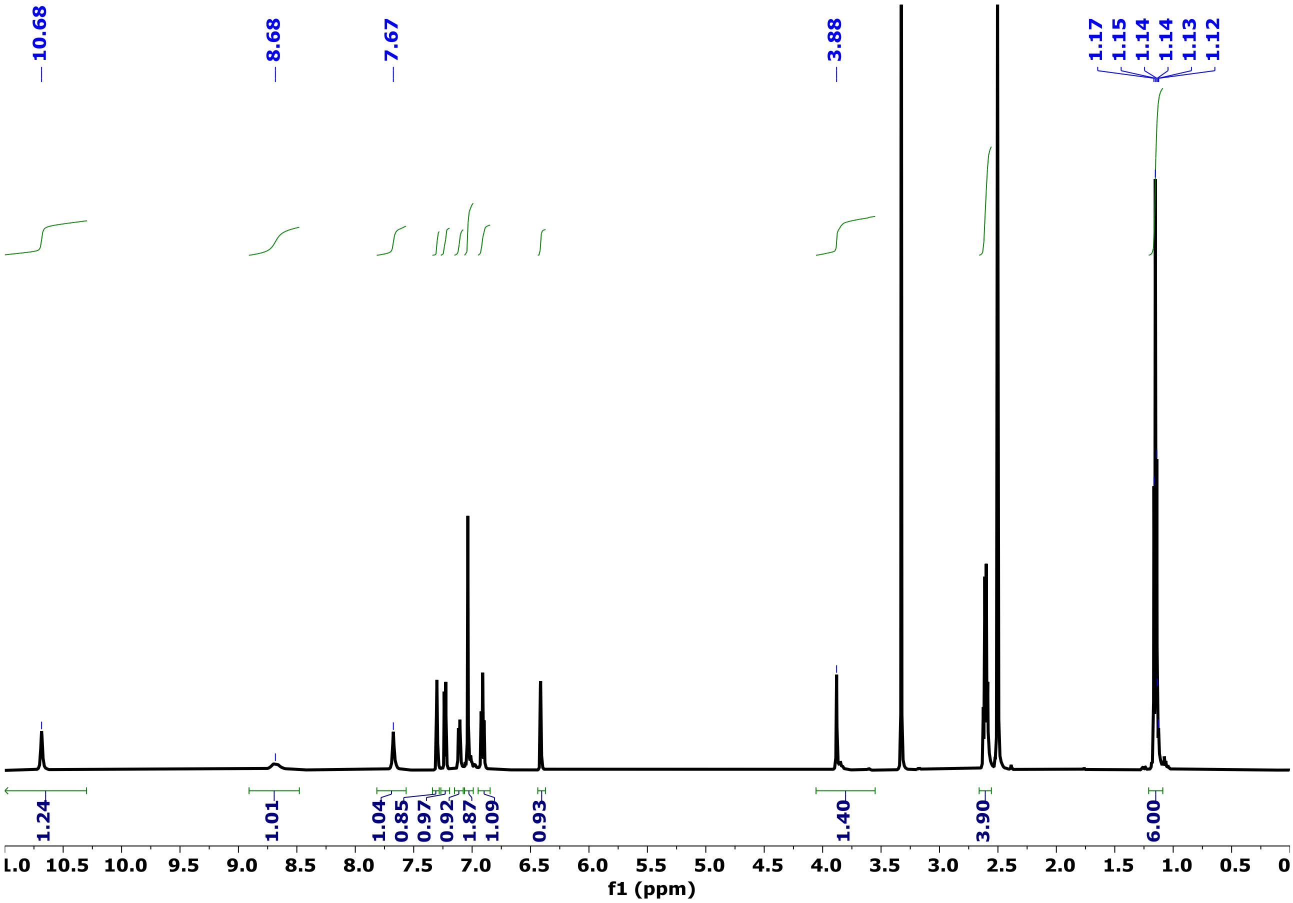


**Figure S1.** $^1$H-NMR spectrum (DMSO-$d_6$) of **L1** at 298 K.

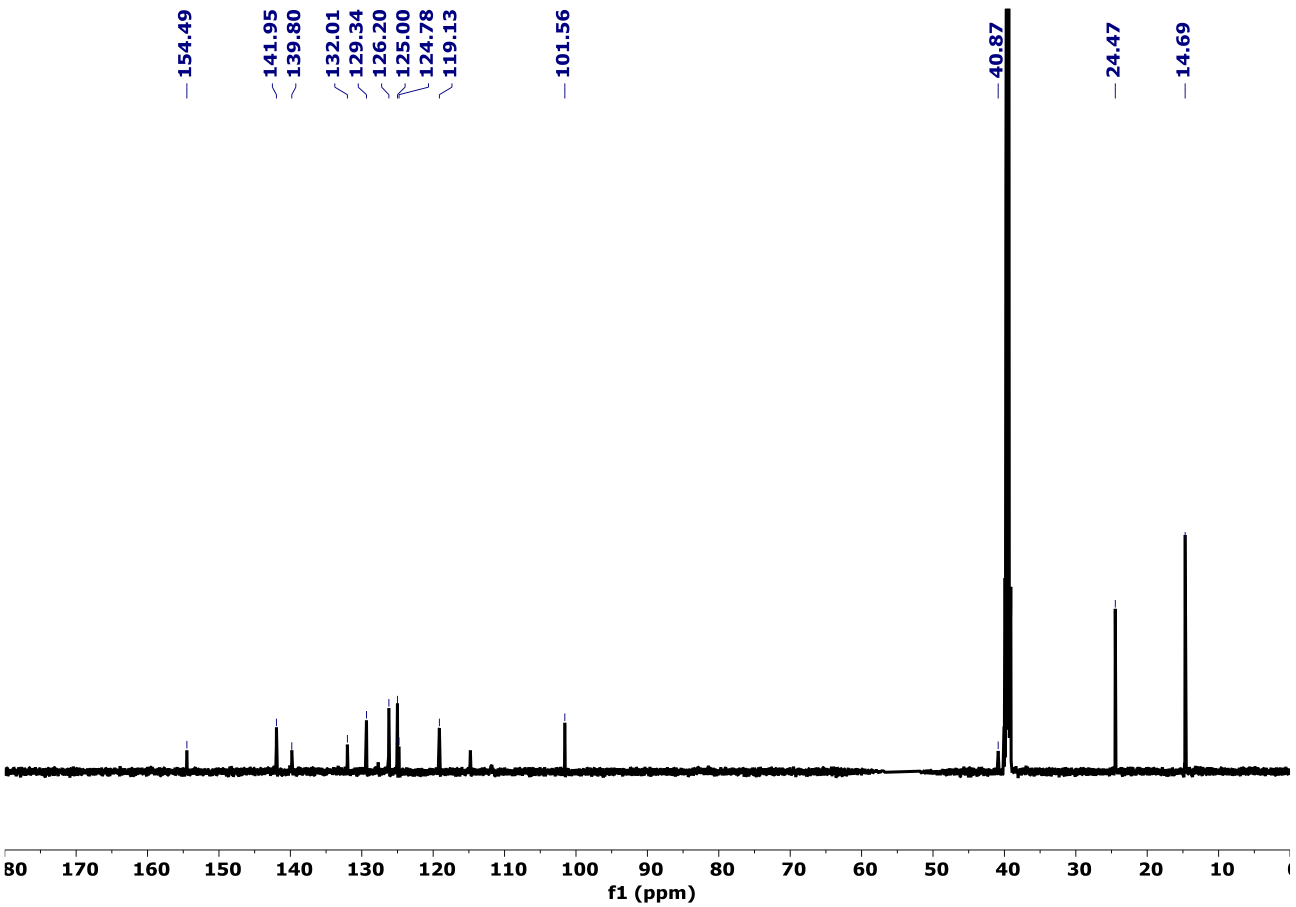


**Figure S2.** $^{13}C$-NMR spectrum (DMSO-$d_6$) of **L1** at 298 K.

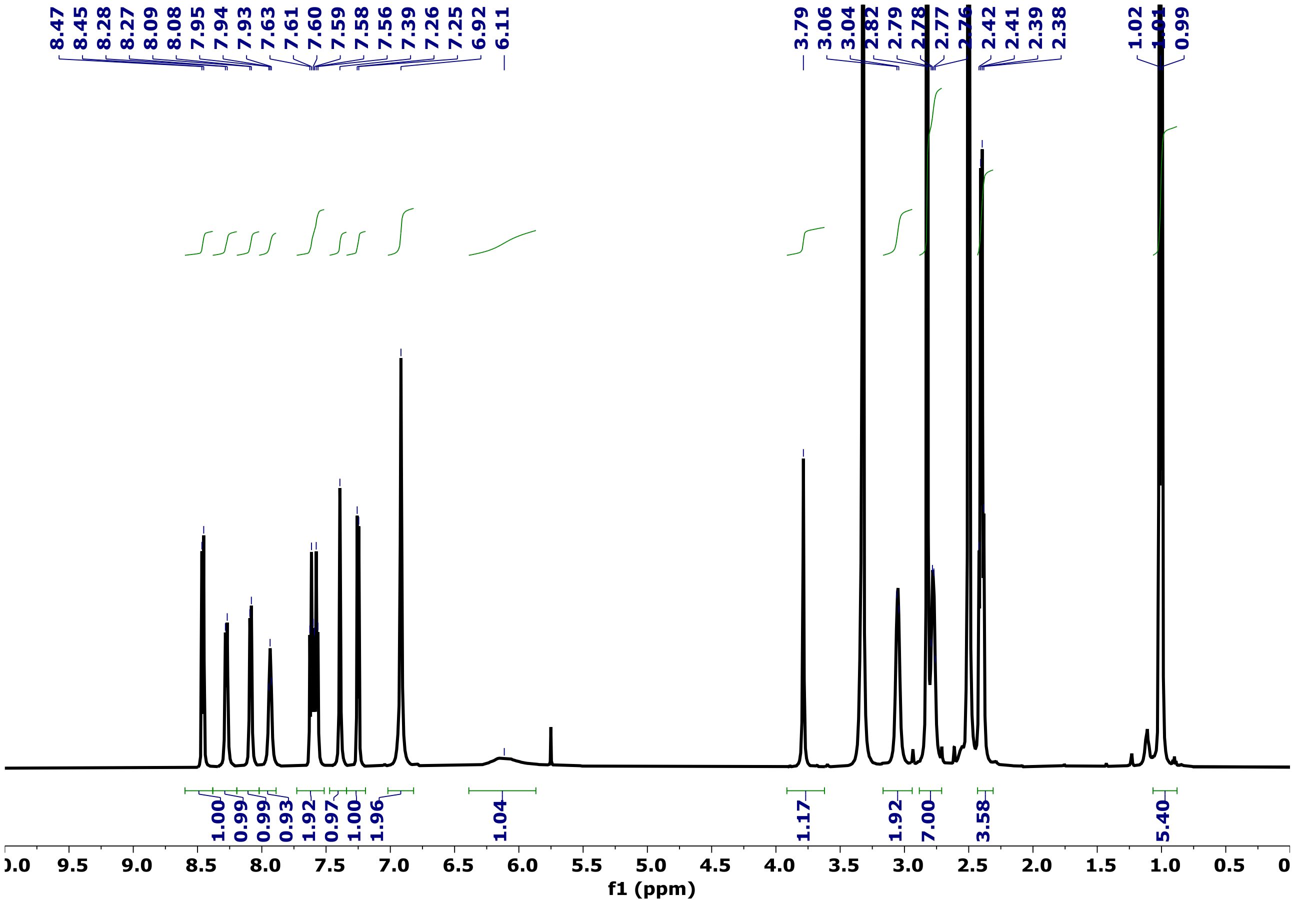


**Figure S3.** $^1$H-NMR spectrum (DMSO-$d_6$) of **L2** at 298 K.

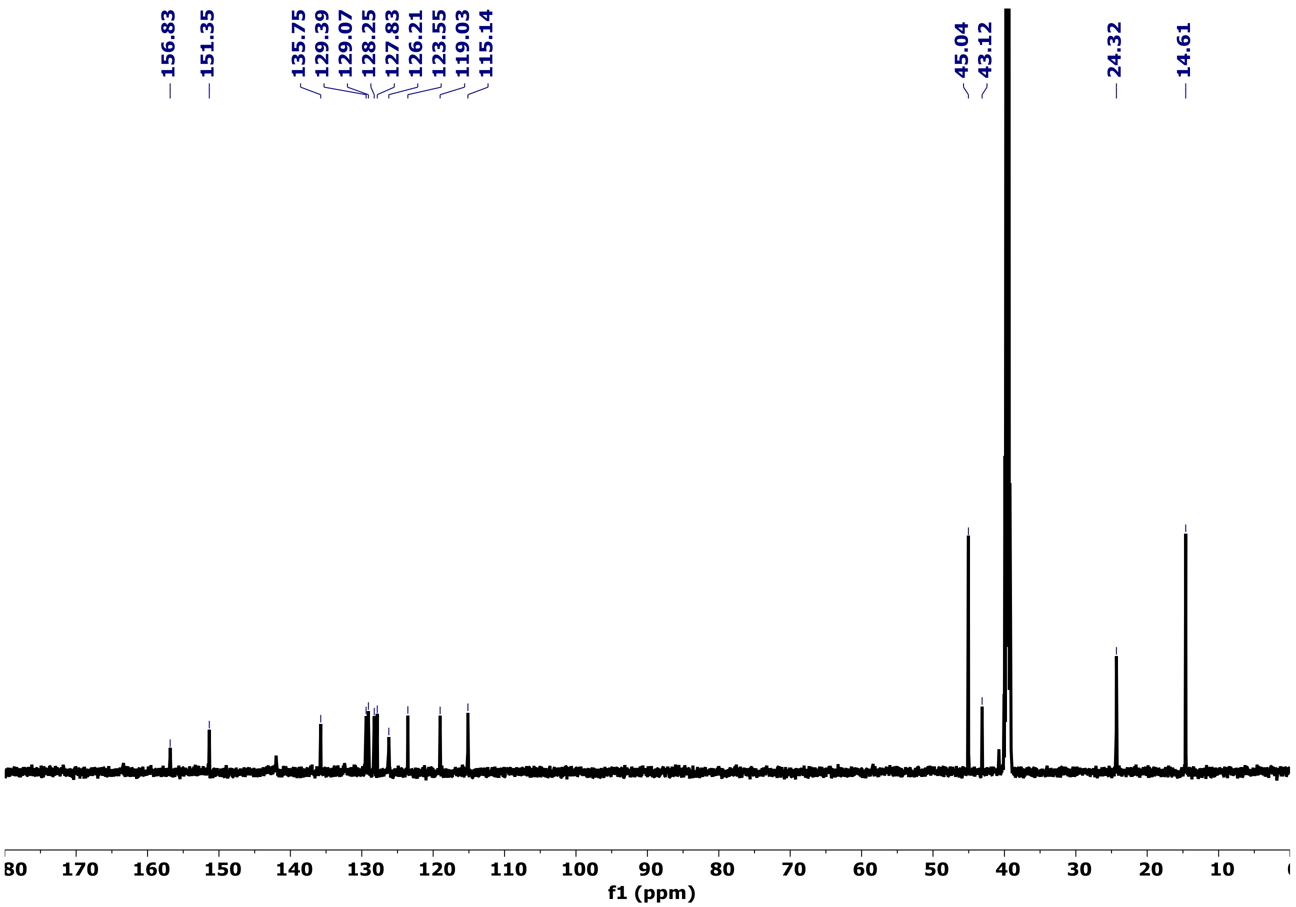


**Figure S4.** $^{13}$C-NMR spectrum (DMSO-$d_6$) of **L2** at 298 K.

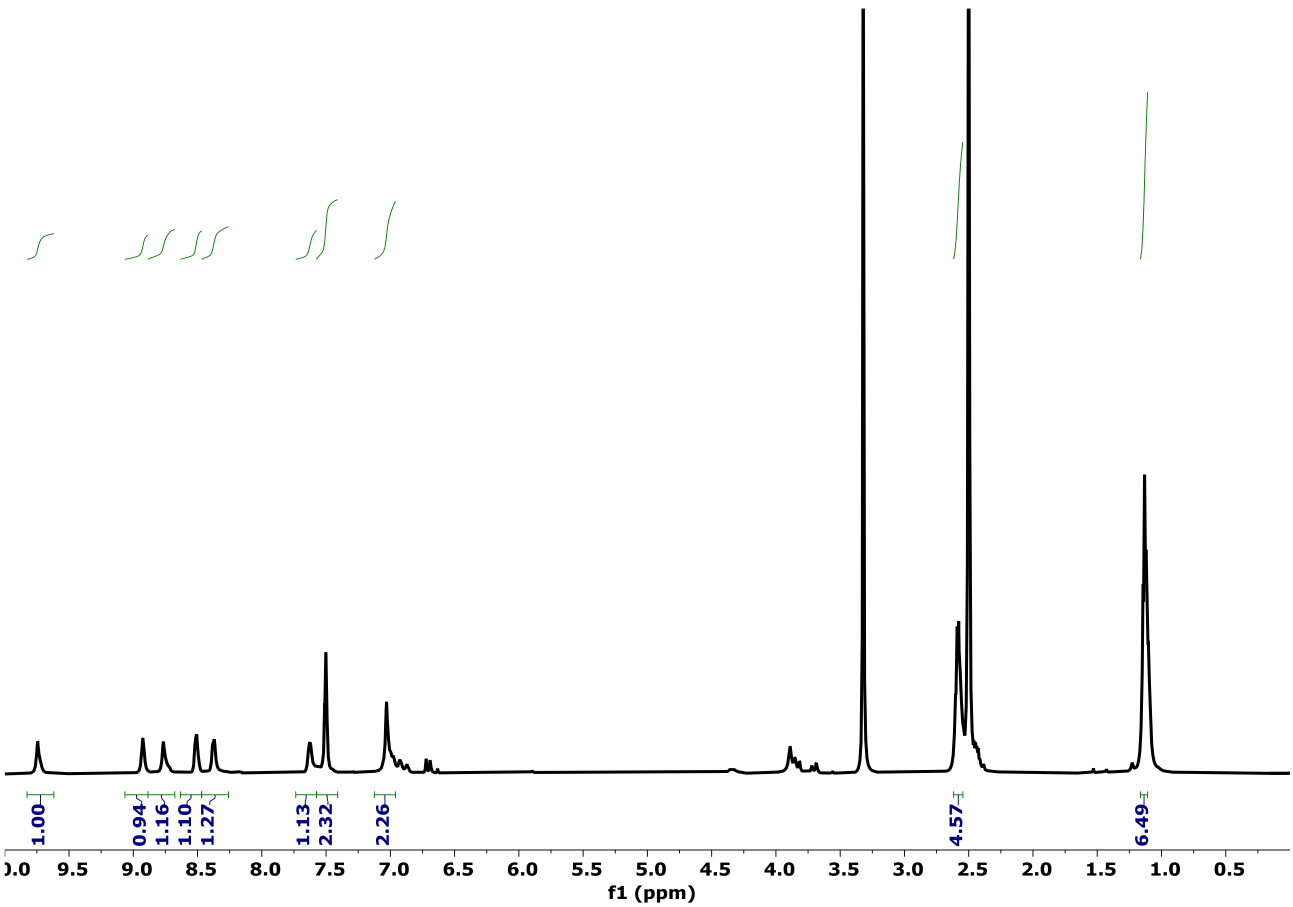


**Figure S5.** $^{1}$H-NMR spectrum (DMSO-$d_6$) of **L3** at 298 K.

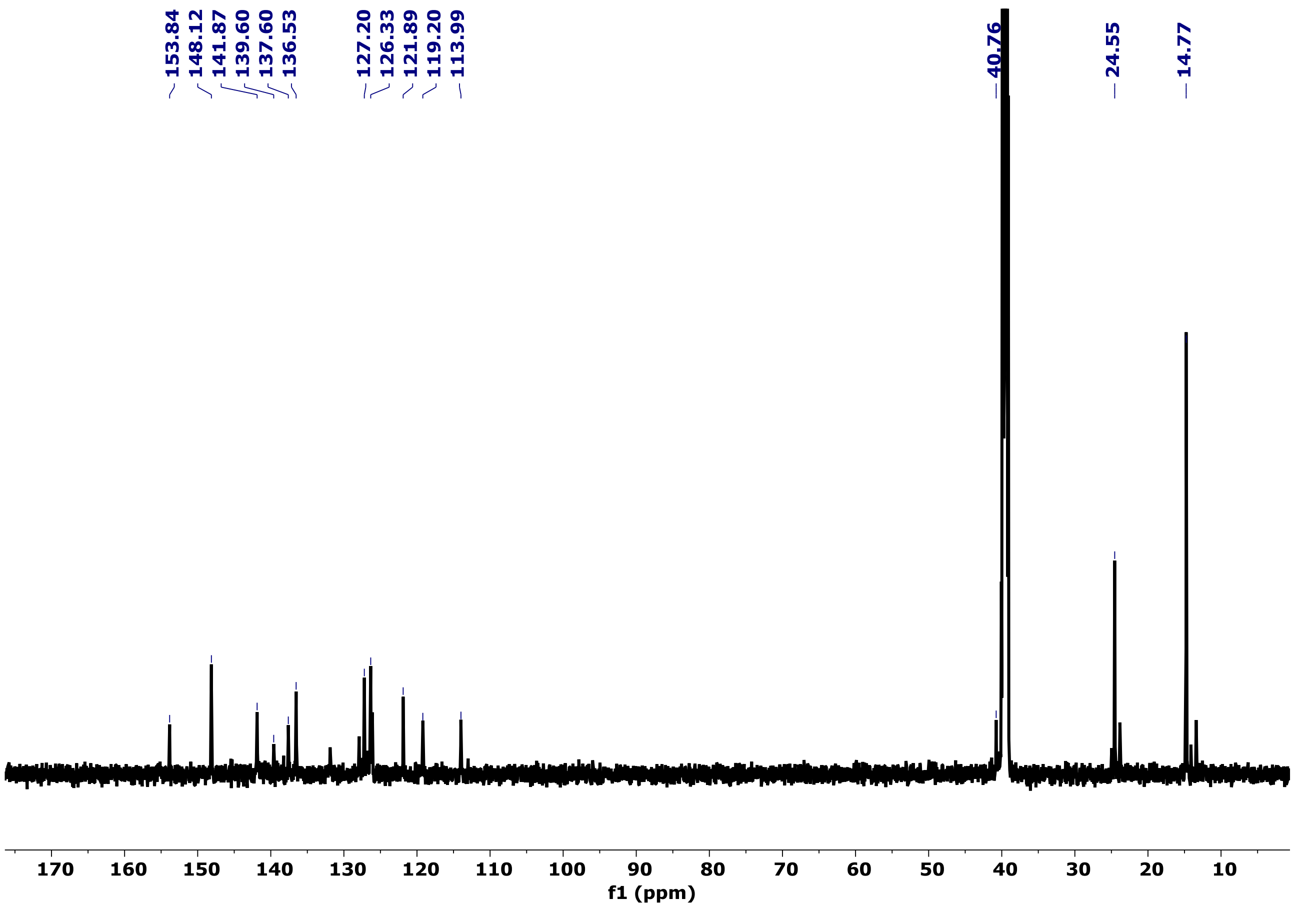


**Figure S6.** $^{13}$C-NMR spectrum (DMSO-$d_6$) of **L3** at 288 K.

The high-resolution mass spectrum for compound **L1** is not reported, as it decomposes in the column.

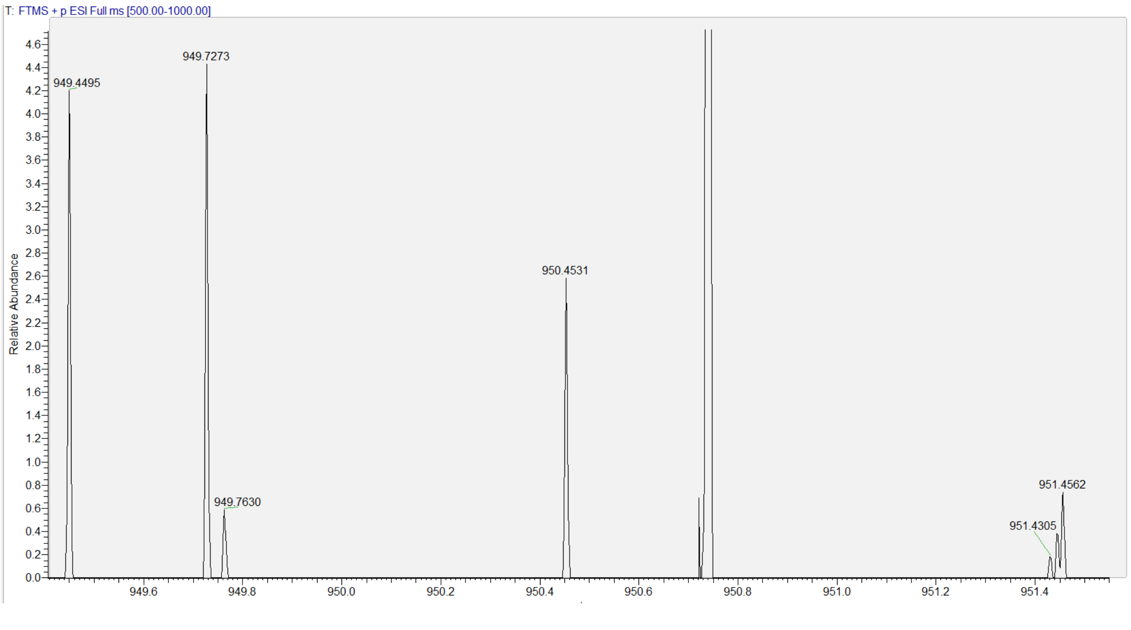

**Figure S7.** High-resolution mass spectrum (ESI+) obtained for compound **L2** in methanol.

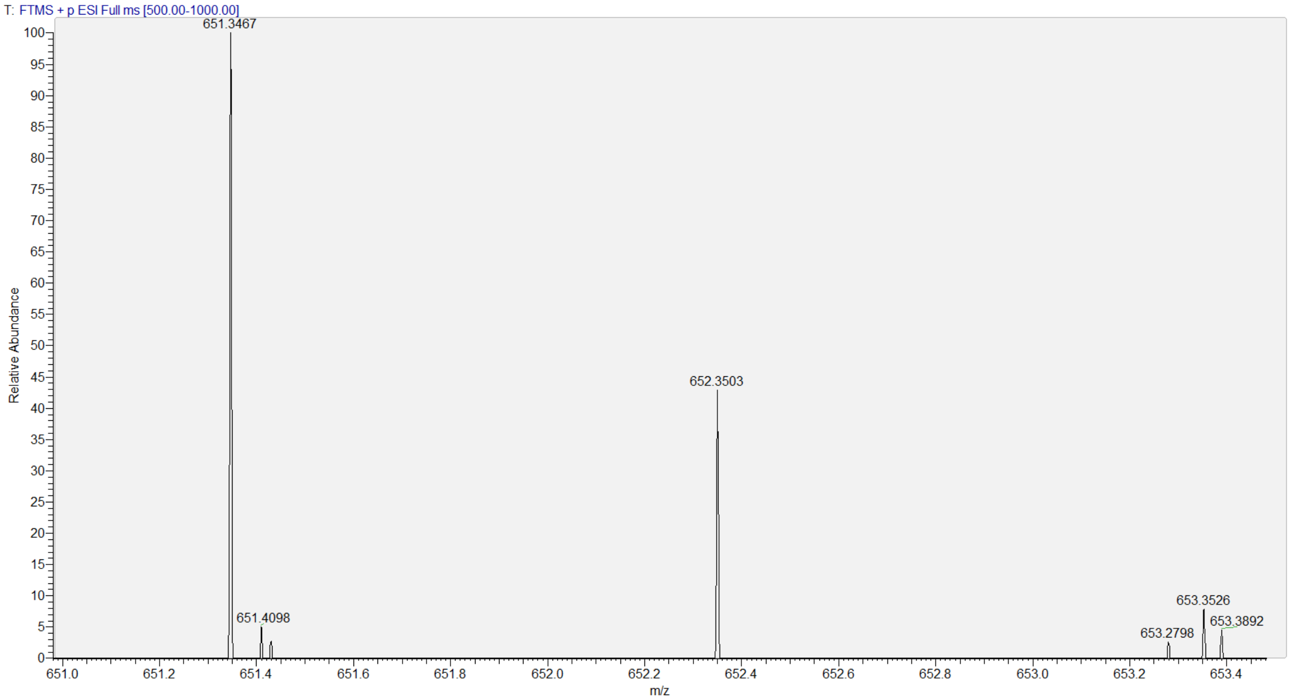


**Figure S8.** High-resolution mass spectrum (ESI+) obtained for compound **L3** in methanol.

## Gels Preparation and Characterisation

### Gel Preparation Procedure:

Gelation tests were carried out at different concentrations in a wide range of solvents (nitrobenzene, chlorobenzene, nitromethane, 1,4-dioxane, tetrahydrofuran, acetonitrile, dichloromethane, chloroform, methanol, ethanol, 1-isopropanol, 2-isopropanol, 1-butanone, ethyl acetate, dimethyl sulfoxide, dimethyl sulfoxide:water). Samples were sonicated, then heated gently to dissolve the solid, and subsequently allowed to cool to room temperature. When a mixture of DMSO/water was used as a solvent, the gelator was dissolved in DMSO. The sample was sonicated in order to completely dissolve the powder. Thus, water was added to the solution of the gelator in DMSO as the antisolvent. The precipitation of the solid was observed. The sample was sonicated, then heated gently to dissolve the solid, and subsequently allowed to cool to room temperature.

### Gel Preparation Procedure in the Presence of Anion Guests:

**L1** was dissolved in DMSO. The sample was sonicated in order to completely dissolve the powder. A water solution of the anion species (as their tetrabutylammonium salts) was prepared in the water fraction of the DMSO/water solvent mixture. Thus, the anion aqueous solution was added to the solution of the gelator in DMSO as the antisolvent. The precipitation of the solid was observed. The sample was sonicated, then heated gently to dissolve the solid, and subsequently allowed to cool to room temperature.

**Table S1.** Gelation tests of **L1-L3** performed at different concentrations with different solvents through a heating and cooling cycle.

| | **L1** | **L2** | **L3** |
|---|---|---|---|
| **$CHCl_3$ (1% w/v)** | insoluble | insoluble | insoluble |
| **DCM (1% w/v)** | insoluble | insoluble | insoluble |
| **Dioxane (1% w/v)** | insoluble | insoluble | insoluble |
| **DMSO (1% w/v)** | no gel | no gel | no gel |
| **DMSO/$H_2O$ (15% $H_2O$) (2% w/v)** | gel | precipitate | precipitate |
| **DMSO/$H_2O$ (20% $H_2O$) (2% w/v)** | gel/precipitate | precipitate | precipitate |
| **EtOAc (1% w/v)** | insoluble | insoluble | insoluble |
| **1-ipOH (1% w/v)** | insoluble | insoluble | insoluble |
| **2-ipOH (1% w/v)** | insoluble | insoluble | insoluble |
| **MeCN (1% w/v)** | insoluble | insoluble | insoluble |
| **MeOH (1% w/v)** | insoluble | insoluble | insoluble |
| **$MeNO_2$ (1% w/v)** | insoluble | insoluble | insoluble |
| **$PhNO_2$ (1% w/v)** | insoluble | gel | no gel |
| **$PhNO_2$ (0.75% w/v)** | insoluble | * | gel |
| **$PhNO_2$ (0.5% w/v)** | insoluble | * | gel |
| **PhCl (1% w/v)** | insoluble | no gel | no gel |
| **PhCl (0.75% w/v)** | insoluble | gel | gel |
| **PhCl (0.5% w/v)** | insoluble | gel | no gel |
| **THF (1% w/v)** | insoluble | insoluble | insoluble |

* Experiments not performed.

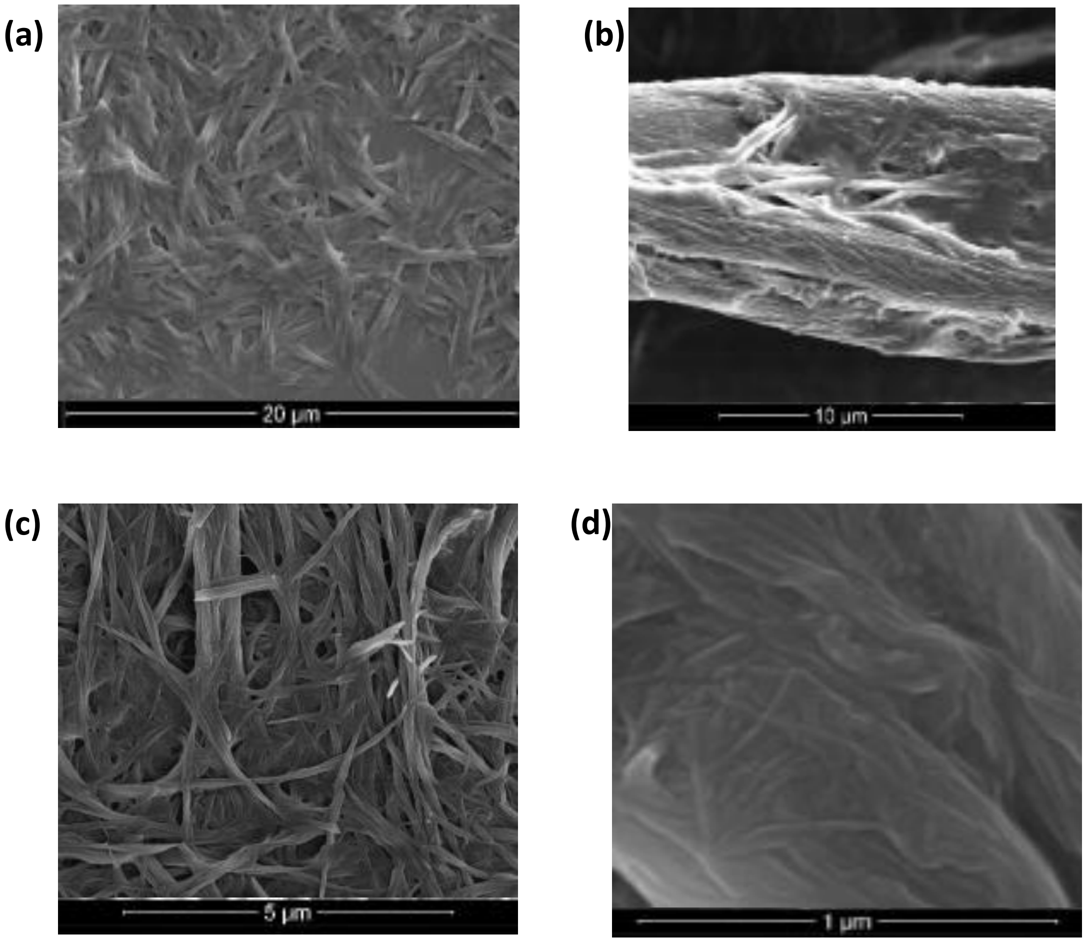


**Figure S9.** SEM images of **L2-L3** xerogels at different concentrations % w/v at different magnifications: (**a**) xerogel of **L3** at 1% w/v in nitrobenzene; (**b**) xerogel of **L3** at 0.75% w/v in chlorobenzene; (**c**) xerogel of **L2** at 0.75% w/v in nitrobenzene; (**d**) ) xerogel of **L2** at 0.75% w/v in chlorobenzene.

## ¹H-NMR titrations

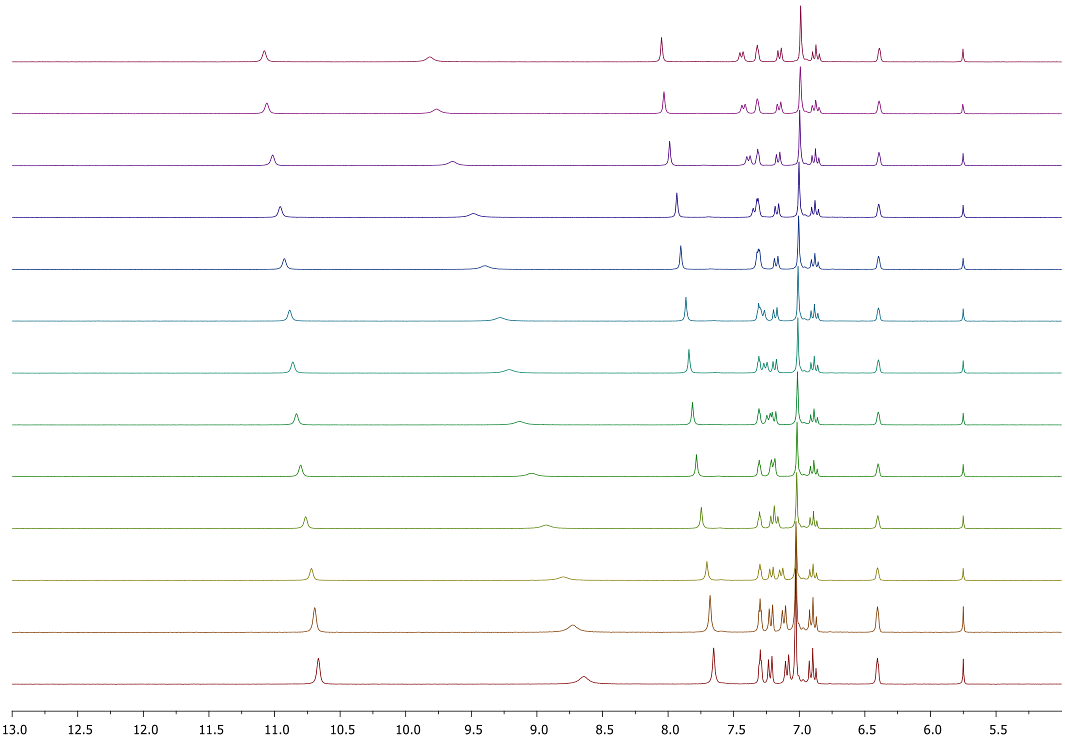


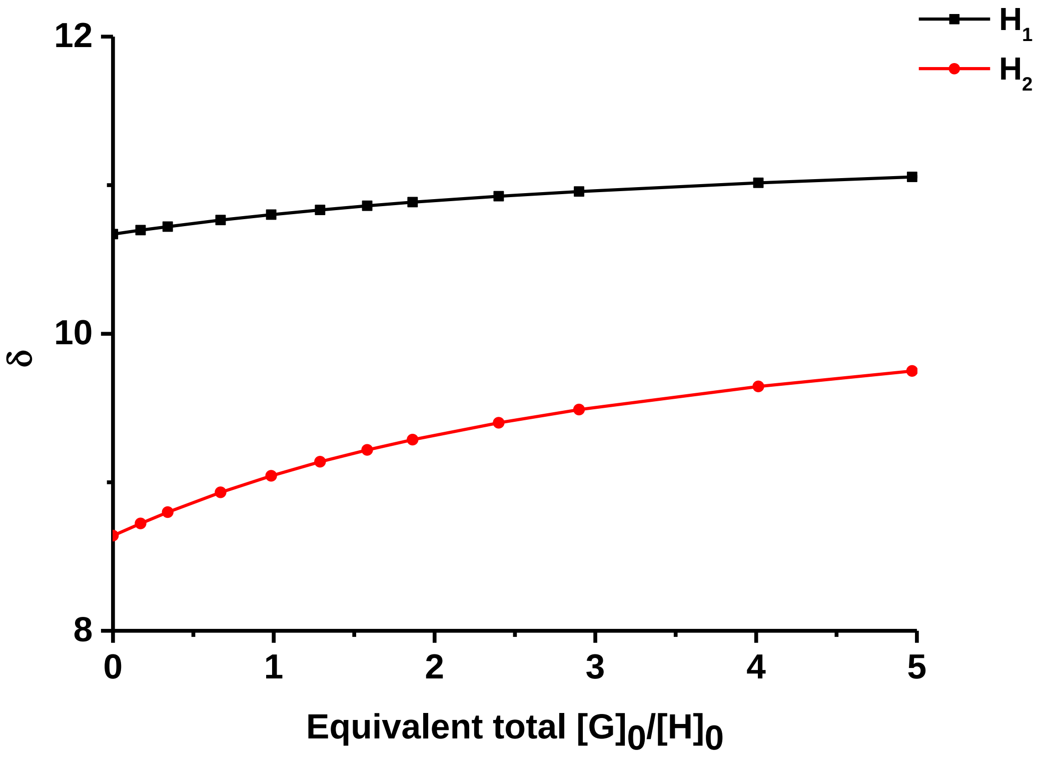

http://app.supramolecular.org/bindfit/view/27cb44e8-1836-490b-8cf4-00bdb6f99620

**Figure S10.** Stack plot of the ¹H-NMR spectra of **L1** (0.005 M) upon addition of increasing amount of TBACl (0.075 M) in DMSO-*d*₆/0.5% water.

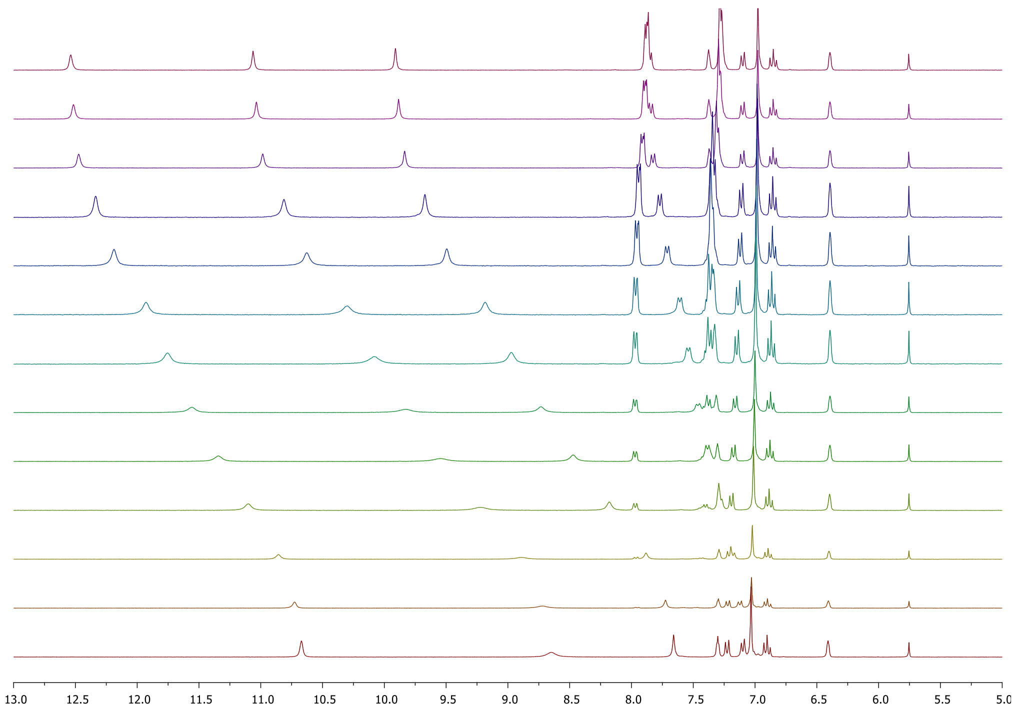


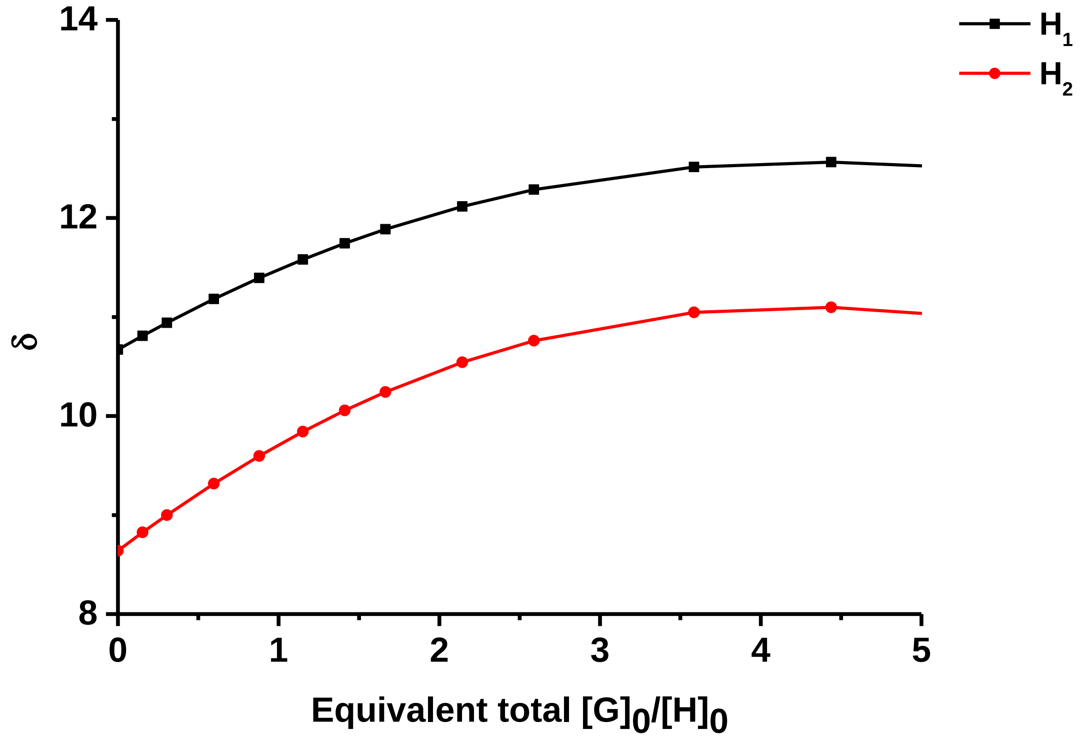


http://app.supramolecular.org/bindfit/view/a88ec1e2-c0bb-475e-9a63-a96b4437b7da

**Figure S11.** Stack plot of the ¹H-NMR spectra of **L1** (0.005 M) upon addition of increasing amount of TBABzO (0.075 M) in DMSO-$d_6$0.5% water.

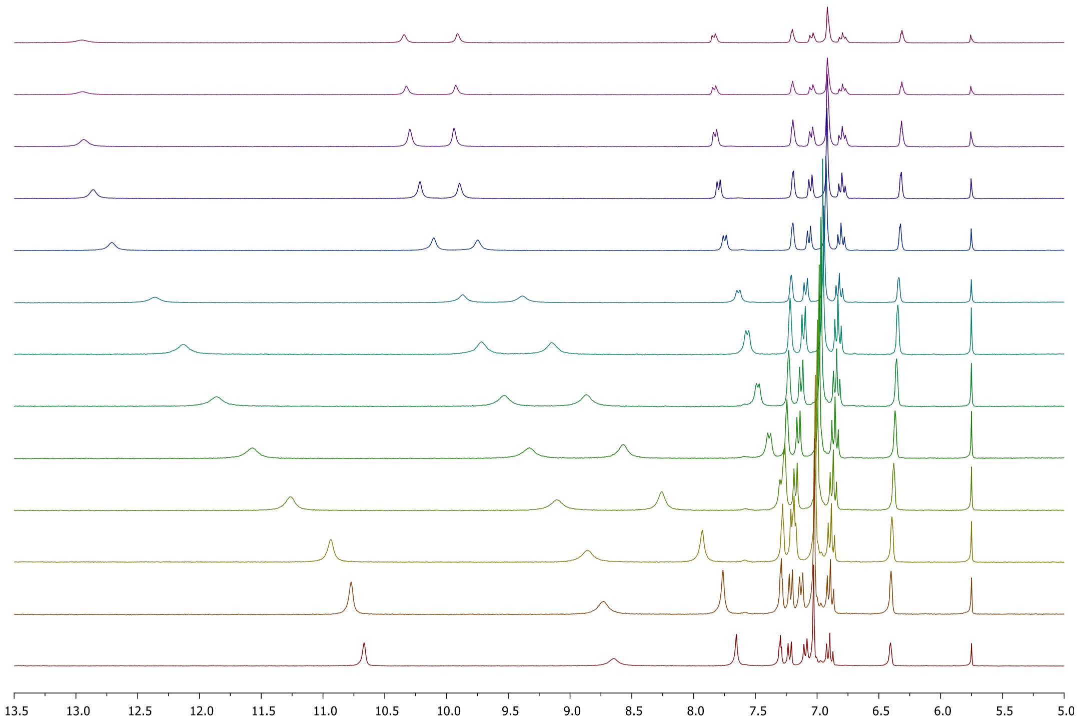


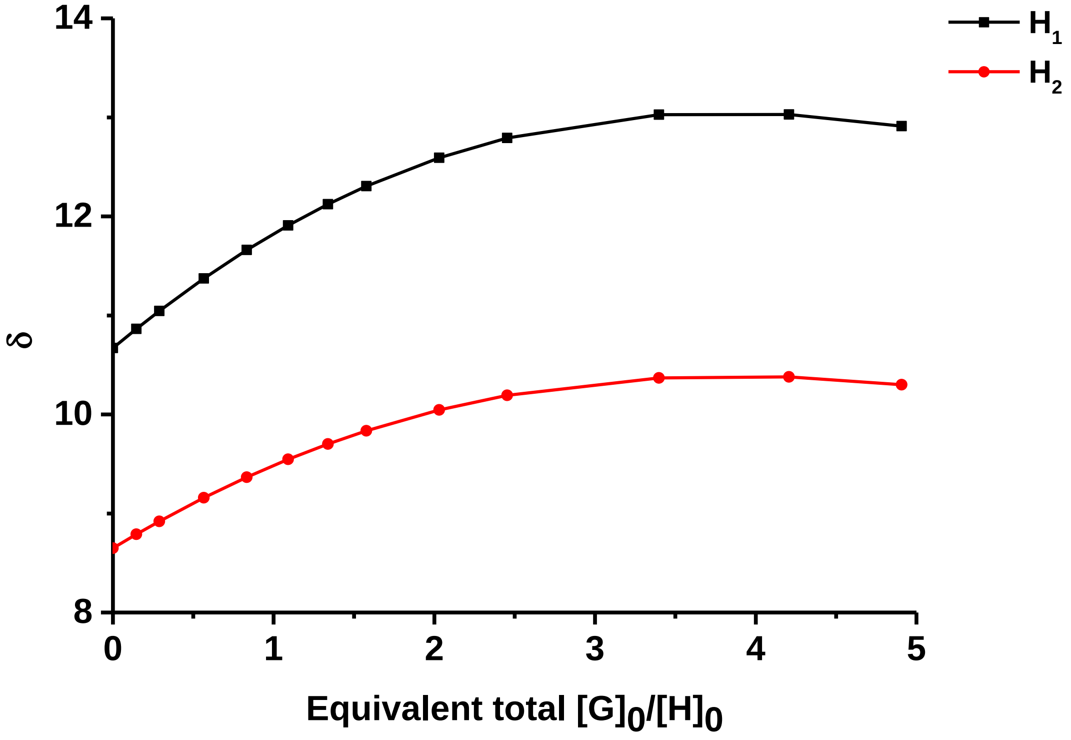

**http://app.supramolecular.org/bindfit/view/89fddba8-cce7-4e44-b0ac-050954774253**

**Figure S12.** Stack plot of the $^{1}H$-NMR spectra of **L1** (0.005 M) upon addition of increasing amount of $TBAH_2PO_4$ (0.075 M) in DMSO-$d_6$/0.5% water.

## DFT Calculations

### *Detail of Additional Guest–Guest and Host–Guest Interaction*

A differentiating feature of the $BzO^-$ complex is the guest–guest interaction. It can be observed in Figure S14a that the aromatic CH bond on one guest points to the π cloud of the other guest with quite a short distance (2.59 Å) to the ring plane. In addition to the extra interaction, there is also a host–guest interaction involving the π cloud of the other guest. That is, an H atom of the ethyl substituents points to the π cloud of the $BzO^-$ anion, thus establishing a CH–π interaction (2.69 Å), as shown in Figure S14b.

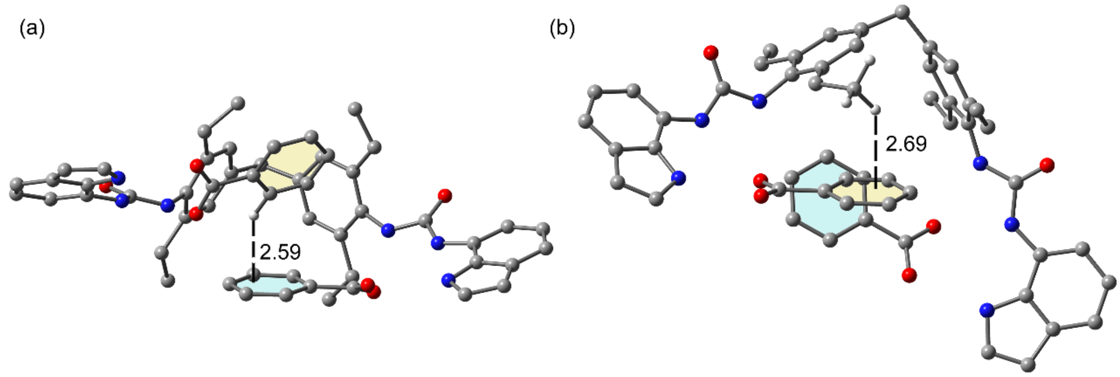


**Figure S13** Two different views of the **L1** host complexed with two $BzO^-$ guests evidencing the T-shape guest–guest stacking (**a**) and the host–guest C–H–π interaction (**b**).

### *Cartesian Coordinates of the Optimised Complexes*

**L1** + 2 $BzO^-$

Energy = -2832.696321909

| | | | |
|---|---|---|---|
| C | -0.4528477 | 0.7533724 | 3.3261942 |
| C | 0.3792527 | 1.8337458 | 2.9382437 |
| C | -0.0184853 | 2.6302350 | 1.8621139 |
| C | -1.2283744 | 2.4048163 | 1.1970476 |
| C | -2.0705132 | 1.3910790 | 1.6606722 |
| C | -1.7005571 | 0.5292710 | 2.7004231 |
| H | 0.6365869 | 3.4258026 | 1.5042980 |
| H | -3.0090292 | 1.2147882 | 1.1346283 |
| C | -1.5163931 | 3.0893534 | -0.1271128 |
| H | -1.0746426 | 4.0961149 | -0.1480601 |
| H | -2.6014772 | 3.1912618 | -0.2786495 |
| C | -0.9089424 | 2.2222795 | -1.2185020 |
| C | 0.3726908 | 2.4844462 | -1.7146402 |
| C | -1.5661676 | 1.0560557 | -1.6253677 |
| C | 1.0336853 | 1.5736942 | -2.5501946 |

| | | | |
|---|---|---|---|
| H | 0.8793637 | 3.3985830 | -1.4019090 |
| C | -0.9492850 | 0.1204098 | -2.4604031 |
| H | -2.5697899 | 0.8575385 | -1.2469271 |
| C | 0.3730320 | 0.3760068 | -2.8809924 |
| C | -2.5570411 | -0.6620209 | 3.0687033 |
| H | -1.8840763 | -1.5246148 | 3.2117044 |
| H | -2.9964300 | -0.4949792 | 4.0643473 |
| C | -3.6555141 | -1.0309164 | 2.0709534 |
| H | -3.2446208 | -1.2038377 | 1.0662418 |
| H | -4.1585738 | -1.9542099 | 2.3906961 |
| H | -4.4223792 | -0.2463918 | 1.9951802 |
| C | 1.6831324 | 2.0706155 | 3.6777990 |
| H | 1.4539242 | 2.1463903 | 4.7547749 |
| H | 2.3219285 | 1.1738413 | 3.5963587 |
| C | 2.4941522 | 3.2878941 | 3.2345135 |
| H | 1.9178866 | 4.2199949 | 3.3322119 |
| H | 3.3969688 | 3.3836430 | 3.8532775 |
| H | 2.8173013 | 3.1998300 | 2.1866195 |
| N | 0.1053328 | -0.1719390 | 4.2350721 |
| H | 1.0570923 | -0.5056200 | 4.0023069 |
| C | -0.4500061 | -0.7490764 | 5.3551073 |
| O | -1.5025554 | -0.3755892 | 5.8943675 |
| N | 0.3374626 | -1.8051011 | 5.7938248 |
| H | 1.1955078 | -1.9538772 | 5.2325364 |
| C | 0.1577371 | -2.6369260 | 6.9012309 |
| C | 0.9235059 | -3.8271309 | 6.9590200 |
| C | -0.7078379 | -2.3876157 | 7.9751105 |
| C | 0.8506203 | -4.7254981 | 8.0666188 |
| C | -0.7923141 | -3.2816887 | 9.0666082 |
| H | -1.3160491 | -1.4871127 | 7.9583959 |
| C | -0.0255688 | -4.4400634 | 9.1365866 |
| C | 1.7585640 | -5.7998578 | 7.7793757 |
| C | 2.3252787 | -5.5276209 | 6.5494297 |
| H | -1.4800998 | -3.0397190 | 9.8793835 |
| H | -0.0998694 | -5.1140979 | 9.9915162 |
| H | 1.9641515 | -6.6650408 | 8.4030522 |
| H | 3.0517610 | -6.0960117 | 5.9753760 |
| N | 1.8321044 | -4.3403474 | 6.0525158 |
| H | 2.0687045 | -3.9870715 | 5.0960482 |
| C | -1.6755747 | -1.1215959 | -2.9374340 |
| H | -2.0255134 | -0.9339459 | -3.9684086 |
| H | -0.9562673 | -1.9450652 | -3.0294867 |
| C | -2.8438580 | -1.5922007 | -2.0706711 |
| H | -2.5069508 | -1.7839257 | -1.0424913 |
| H | -3.6645070 | -0.8611106 | -2.0361957 |
| H | -3.2505536 | -2.5320025 | -2.4681182 |
| C | 2.4300625 | 1.8226955 | -3.0792552 |
| H | 3.0189388 | 0.9035426 | -2.9343918 |
| H | 2.3623411 | 1.9361621 | -4.1748156 |
| C | 3.1771739 | 3.0124193 | -2.4773764 |
| H | 2.6704124 | 3.9663093 | -2.6863321 |
| H | 3.2739219 | 2.9161211 | -1.3853553 |
| H | 4.1897914 | 3.0760632 | -2.8993454 |
| N | 1.0778638 | -0.6447482 | -3.5872585 |
| C | 1.0839267 | -0.7376195 | -4.9613682 |
| O | 0.7997938 | 0.2070708 | -5.7142369 |
| N | 1.4495890 | -2.0061542 | -5.3864119 |
| H | 1.5253446 | -2.7093768 | -4.6259984 |
| H | 1.1964074 | -1.5409881 | -3.0855032 |
| C | 1.5813262 | -2.4593495 | -6.7030072 |
| C | 1.4049029 | -3.8391087 | -6.9616080 |
| C | 1.9157300 | -1.6473587 | -7.7949154 |
| C | 1.5842906 | -4.3965713 | -8.2652525 |
| C | 2.0768540 | -2.1907039 | -9.0888241 |
| H | 2.0519576 | -0.5809625 | -7.6308413 |

| | | | |
|---|---|---|---|
| C | 1.9252248 | -3.5503231 | -9.3421762 |
| C | 1.3291591 | -5.8040644 | -8.1441706 |
| C | 1.0105073 | -6.0405415 | -6.8210985 |
| H | 2.3384735 | -1.5147586 | -9.9054347 |
| H | 2.0615222 | -3.9541863 | -10.3468288 |
| H | 1.3744263 | -6.5463754 | -8.9360081 |
| N | 1.0613943 | -4.8655385 | -6.1023646 |
| H | 0.7542022 | -4.8022571 | -5.1011647 |
| H | 0.7423088 | -6.9687376 | -6.3241070 |
| C | 2.8663250 | -2.0765301 | 1.6920338 |
| C | 3.4572619 | -2.9775352 | 0.7924539 |
| C | 3.7014098 | -2.6033781 | -0.5299168 |
| C | 3.3332449 | -1.3301625 | -0.9776520 |
| C | 2.7184009 | -0.4336965 | -0.0973270 |
| C | 2.4988949 | -0.8030824 | 1.2318982 |
| H | 3.7182327 | -3.9760571 | 1.1451391 |
| H | 4.1509727 | -3.3134638 | -1.2244478 |
| H | 3.4894130 | -1.0552373 | -2.0209396 |
| H | 2.3823211 | 0.5422442 | -0.4493214 |
| H | 2.0134134 | -0.1045853 | 1.9110618 |
| C | 2.6178400 | -2.4815015 | 3.1289393 |
| O | 2.4785379 | -1.5414071 | 3.9878095 |
| O | 2.5634538 | -3.7124241 | 3.4119010 |
| C | -0.2249757 | -3.9583041 | -1.3661308 |
| C | 0.1266379 | -2.9139643 | -0.4974934 |
| C | -0.5293415 | -2.7506293 | 0.7230134 |
| C | -1.5389602 | -3.6428125 | 1.0961700 |
| C | -1.9022867 | -4.6851274 | 0.2353559 |
| C | -1.2531424 | -4.8369266 | -0.9919774 |
| H | 0.9181771 | -2.2263955 | -0.7874651 |
| H | -0.2502586 | -1.9243511 | 1.3787201 |
| H | -2.0506820 | -3.5248639 | 2.0519341 |
| H | -2.6969356 | -5.3767751 | 0.5210389 |
| H | -1.5349975 | -5.6376401 | -1.6767340 |
| C | 0.4742015 | -4.1158178 | -2.7021837 |
| O | 0.0314237 | -4.9831522 | -3.5151686 |
| O | 1.4664474 | -3.3490537 | -2.9430005 |

## **L1** + 2 $H_2PO_4^-$

Energy = -3279.499290098

| | | | |
|---|---|---|---|
| C | -0.1680206 | 0.3083554 | 3.6326044 |
| C | 0.8634533 | 1.1179503 | 3.1111449 |
| C | 0.6049598 | 1.8339133 | 1.9327730 |
| C | -0.6201391 | 1.7426219 | 1.2657195 |
| C | -1.6020803 | 0.8826043 | 1.7757608 |
| C | -1.4010147 | 0.1591701 | 2.9547445 |
| H | 1.3807027 | 2.4775959 | 1.5154826 |
| H | -2.5526880 | 0.8008316 | 1.2476019 |
| C | -0.8747123 | 2.5382776 | -0.0015033 |
| H | -0.2384964 | 3.4347542 | 0.0004883 |
| H | -1.9201828 | 2.8840757 | -0.0043800 |
| C | -0.6140829 | 1.7437636 | -1.2682122 |
| C | 0.6204870 | 1.8220332 | -1.9198655 |
| C | -1.6003889 | 0.8976508 | -1.7923016 |
| C | 0.8836577 | 1.1065913 | -3.0970827 |
| H | 1.3996611 | 2.4542604 | -1.4913095 |
| C | -1.3936394 | 0.1737531 | -2.9703902 |
| H | -2.5582347 | 0.8262224 | -1.2755555 |
| C | -0.1514660 | 0.3111337 | -3.6321105 |
| C | -2.4639402 | -0.7497790 | 3.5415942 |
| H | -2.0457811 | -1.7660167 | 3.6472296 |
| H | -2.6613158 | -0.4234908 | 4.5773951 |
| C | -3.7812388 | -0.8319472 | 2.7696104 |

| | | | |
|---|---|---|---|
| H | -3.6310682 | -1.2168570 | 1.7496938 |
| H | -4.4760907 | -1.5110685 | 3.2826189 |
| H | -4.2694065 | 0.1505693 | 2.6909155 |
| C | 2.2080790 | 1.1856853 | 3.8026295 |
| H | 2.1212633 | 1.8581078 | 4.6715963 |
| H | 2.4119942 | 0.1928289 | 4.2362546 |
| C | 3.3870117 | 1.6177064 | 2.9265887 |
| H | 3.2920209 | 2.6594298 | 2.5871961 |
| H | 4.3251656 | 1.5439802 | 3.4945520 |
| H | 3.4793366 | 0.9812463 | 2.0338291 |
| N | 0.0174009 | -0.4076549 | 4.8410245 |
| H | -0.2327949 | -1.4093987 | 4.8649148 |
| C | 0.1794132 | 0.1981903 | 6.0687578 |
| O | 0.2822486 | 1.4238087 | 6.2265287 |
| N | 0.2335191 | -0.7281279 | 7.0988680 |
| H | 0.0969205 | -1.7052754 | 6.8069946 |
| C | 0.2965387 | -0.4721573 | 8.4760512 |
| C | 0.7550125 | -1.5035783 | 9.3276195 |
| C | -0.0946441 | 0.7256796 | 9.0887936 |
| C | 0.7932397 | -1.3599017 | 10.7485494 |
| C | -0.0409525 | 0.8807159 | 10.4920521 |
| H | -0.4464961 | 1.5431761 | 8.4651426 |
| C | 0.3870121 | -0.1414515 | 11.3331100 |
| C | 1.3021382 | -2.5981277 | 11.2678129 |
| C | 1.5515007 | -3.4160866 | 10.1831259 |
| H | -0.3585350 | 1.8343887 | 10.9184074 |
| H | 0.4146843 | -0.0046040 | 12.4154122 |
| H | 1.4648468 | -2.8508715 | 12.3117180 |
| H | 1.9491526 | -4.4265624 | 10.1485407 |
| N | 1.2186406 | -2.7651359 | 9.0144522 |
| H | 1.4020870 | -3.1480507 | 8.0621100 |
| C | -2.4572359 | -0.7254689 | -3.5701519 |
| H | -2.6305200 | -0.4054279 | -4.6120071 |
| H | -2.0497992 | -1.7480193 | -3.6568783 |
| C | -3.7892898 | -0.7842630 | -2.8218613 |
| H | -3.6623862 | -1.1653724 | -1.7976020 |
| H | -4.2639716 | 0.2059013 | -2.7574781 |
| H | -4.4844598 | -1.4568398 | -3.3432684 |
| C | 2.2349653 | 1.1577004 | -3.7768004 |
| H | 2.4317760 | 0.1609174 | -4.2054924 |
| H | 2.1633030 | 1.8294980 | -4.6480306 |
| C | 3.4110464 | 1.5779598 | -2.8917611 |
| H | 3.3247636 | 2.6210206 | -2.5537142 |
| H | 3.4886170 | 0.9407718 | -1.9983875 |
| H | 4.3528251 | 1.4929884 | -3.4521620 |
| N | 0.0422043 | -0.4061917 | -4.8388259 |
| C | 0.2257652 | 0.1914763 | -6.0651390 |
| O | 0.3083665 | 1.4170368 | -6.2321932 |
| N | 0.3237817 | -0.7433445 | -7.0874623 |
| H | 0.2184471 | -1.7197478 | -6.7873024 |
| H | -0.1666282 | -1.4188024 | -4.8563183 |
| C | 0.3532008 | -0.4994804 | -8.4686909 |
| C | 0.7964204 | -1.5351706 | -9.3235003 |
| C | -0.0605844 | 0.6907066 | -9.0821545 |
| C | 0.7953814 | -1.4033705 | -10.7462878 |
| C | -0.0443266 | 0.8341951 | -10.4873160 |
| H | -0.4022618 | 1.5113456 | -8.4571298 |
| C | 0.3666442 | -0.1926423 | -11.3305399 |
| C | 1.2945780 | -2.6433704 | -11.2700542 |
| C | 1.5763246 | -3.4520715 | -10.1865892 |
| H | -0.3785966 | 1.7823661 | -10.9128757 |
| H | 0.3643853 | -0.0664766 | -12.4146714 |
| H | 1.4299621 | -2.9025113 | -12.3162445 |
| N | 1.2725799 | -2.7934391 | -9.0143446 |
| H | 1.4891956 | -3.1737412 | -8.0689035 |

| | | | |
|---|---|---|---|
| H | 1.9795345 | -4.4603191 | -10.1540661 |
| P | 0.7334966 | -3.9934368 | 5.4905930 |
| O | 1.8211709 | -3.8511952 | 6.5286204 |
| O | 0.2940910 | -5.5661132 | 5.5237477 |
| H | -0.4629598 | -5.7324249 | 4.9302711 |
| O | 1.3308609 | -3.7669685 | 3.9898485 |
| H | 2.2088552 | -4.1825238 | 3.8952996 |
| O | -0.4920987 | -3.1054877 | 5.5712655 |
| P | 1.0942969 | -3.8974022 | -5.3784067 |
| O | 1.9848723 | -3.9383395 | -6.5921325 |
| O | 2.0180836 | -3.3126338 | -4.1623910 |
| H | 1.4726733 | -3.0335199 | -3.4039223 |
| O | 0.8024879 | -5.4501807 | -4.9704555 |
| H | 0.0651220 | -5.5145870 | -4.3343997 |
| O | -0.2179650 | -3.1341180 | -5.4263169 |

Trimer (**L1**)$_3$

| | | | |
|---|---|---|---|
| C | 2.4221626 | -4.8219048 | 3.5310698 |
| C | 3.5013294 | -4.0270179 | 3.1039837 |
| C | 3.3236602 | -3.2388744 | 1.9624405 |
| C | 2.1115419 | -3.2254492 | 1.2676539 |
| C | 1.0586115 | -4.0259909 | 1.7218551 |
| C | 1.1919017 | -4.8399724 | 2.8518520 |
| H | 4.1363320 | -2.6035910 | 1.6096049 |
| H | 0.1150213 | -4.0079185 | 1.1757467 |
| C | 1.9572874 | -2.3989480 | 0.0115251 |
| H | 2.6404319 | -1.5399209 | 0.0493085 |
| H | 0.9434424 | -1.9804261 | -0.0359172 |
| C | 2.2187169 | -3.2129486 | -1.2357703 |
| C | 3.5184813 | -3.3821356 | -1.7236668 |
| C | 1.1596806 | -3.8333600 | -1.9011533 |
| C | 3.7742924 | -4.1223129 | -2.8825466 |
| H | 4.3423248 | -2.9035569 | -1.1929052 |
| C | 1.3657676 | -4.6011381 | -3.0508160 |
| H | 0.1480563 | -3.6912217 | -1.5201098 |
| C | 2.6816950 | -4.7196320 | -3.5366280 |
| C | 0.0719936 | -5.7170152 | 3.3652635 |
| H | 0.5013846 | -6.6989550 | 3.6198217 |
| H | -0.2782977 | -5.3004258 | 4.3253776 |
| C | -1.1108281 | -5.9036368 | 2.4172499 |
| H | -0.7845957 | -6.3070244 | 1.4464391 |
| H | -1.8378149 | -6.6058534 | 2.8475733 |
| H | -1.6389083 | -4.9582097 | 2.2268021 |
| C | 4.7938332 | -4.0393766 | 3.8909503 |
| H | 4.6023383 | -3.5894397 | 4.8811367 |
| H | 5.0581071 | -5.0877795 | 4.1036003 |
| C | 5.9831848 | -3.3419244 | 3.2324468 |
| H | 5.8053405 | -2.2667231 | 3.0903960 |
| H | 6.8791768 | -3.4474036 | 3.8587531 |
| H | 6.2066086 | -3.7754523 | 2.2457705 |
| N | 2.5942942 | -5.6302888 | 4.7004329 |
| H | 3.0387959 | -6.5464131 | 4.6197927 |
| C | 2.2249168 | -5.1735809 | 5.9286536 |
| O | 1.5374040 | -4.1281290 | 6.0490268 |
| N | 2.6606141 | -5.9104600 | 7.0003478 |
| H | 2.9388103 | -6.8774260 | 6.7740006 |
| C | 2.1740756 | -5.6896150 | 8.3153787 |
| C | 1.4080320 | -6.6791116 | 8.9490236 |
| C | 2.4226898 | -4.5128522 | 9.0188343 |
| C | 0.8502238 | -6.4911790 | 10.2478319 |
| C | 1.8714531 | -4.3065575 | 10.3003689 |
| H | 3.0244761 | -3.7344742 | 8.5490568 |
| C | 1.0816525 | -5.2732886 | 10.9155828 |
| C | 0.1276246 | -7.6941822 | 10.5545682 |
| C | 0.2834632 | -8.5459433 | 9.4812356 |

| | | | |
|---|---|---|---|
| H | 2.0454905 | -3.3525277 | 10.7989502 |
| H | 0.6377976 | -5.0846302 | 11.8940059 |
| H | -0.4195197 | -7.9138625 | 11.4662566 |
| H | -0.0600412 | -9.5661194 | 9.3421464 |
| N | 1.0342357 | -7.9272076 | 8.5008878 |
| H | 1.4363825 | -8.3822523 | 7.6790455 |
| C | 0.2274894 | -5.2816652 | -3.7781547 |
| H | 0.0312869 | -4.7365132 | -4.7185842 |
| H | 0.5739370 | -6.2788452 | -4.0924432 |
| C | -1.0759209 | -5.4102895 | -2.9905877 |
| H | -0.9151208 | -5.9220586 | -2.0293756 |
| H | -1.5232382 | -4.4306003 | -2.7736016 |
| H | -1.8128745 | -5.9885325 | -3.5640774 |
| C | 5.1598440 | -4.2591405 | -3.4749507 |
| H | 5.3483382 | -5.3247090 | -3.6824978 |
| H | 5.1558981 | -3.7721963 | -4.4656689 |
| C | 6.3024436 | -3.7013914 | -2.6292933 |
| H | 6.2023979 | -2.6181534 | -2.4682973 |
| H | 6.3427501 | -4.1853706 | -1.6416974 |
| H | 7.2657942 | -3.8746512 | -3.1281556 |
| N | 2.9047742 | -5.4717725 | -4.7295389 |
| C | 2.5591907 | -4.9791880 | -5.9522466 |
| O | 2.2320788 | -3.7750295 | -6.1060360 |
| N | 2.5772504 | -5.8856679 | -6.9817411 |
| H | 3.0717806 | -6.7646887 | -6.7669279 |
| H | 3.3647481 | -6.3856657 | -4.6848677 |
| C | 2.5848256 | -5.4812254 | -8.3426098 |
| C | 3.7365301 | -5.6887506 | -9.1153212 |
| C | 1.4901755 | -4.8807738 | -8.9590768 |
| C | 3.8299454 | -5.2573240 | -10.4704078 |
| C | 1.5656244 | -4.4427781 | -10.2979855 |
| H | 0.5795587 | -4.7240992 | -8.3803158 |
| C | 2.7227487 | -4.6107978 | -11.0528640 |
| C | 5.1413838 | -5.6287928 | -10.9232380 |
| C | 5.7673005 | -6.2692183 | -9.8748935 |
| H | 0.7052291 | -3.9327124 | -10.7321603 |
| H | 2.7743837 | -4.2381313 | -12.0766529 |
| H | 5.5610458 | -5.4688840 | -11.9117991 |
| N | 4.9301171 | -6.2875896 | -8.7755478 |
| H | 5.0824075 | -6.8248072 | -7.9224502 |
| H | 6.7412744 | -6.7455118 | -9.8267763 |
| C | 1.4784099 | -0.7834692 | 3.5814767 |
| C | 2.4940126 | -0.0165362 | 2.9911269 |
| C | 2.1959781 | 0.6766629 | 1.8089047 |
| C | 0.9301488 | 0.6066124 | 1.2201656 |
| C | -0.0428498 | -0.2154329 | 1.8043093 |
| C | 0.2098028 | -0.9211781 | 2.9842243 |
| H | 2.9632098 | 1.2892321 | 1.3325161 |
| H | -1.0172551 | -0.2979946 | 1.3207089 |
| C | 0.6187057 | 1.3820927 | -0.0484013 |
| H | 1.3757475 | 2.1689207 | -0.1814852 |
| H | -0.3527938 | 1.8868978 | 0.0701489 |
| C | 0.5745410 | 0.5122387 | -1.2911895 |
| C | 1.7432388 | 0.2552723 | -2.0208615 |
| C | -0.6219223 | -0.0740414 | -1.7120033 |
| C | 1.7264176 | -0.5313345 | -3.1755107 |
| H | 2.6818860 | 0.6910722 | -1.6749974 |
| C | -0.6861061 | -0.8822789 | -2.8566262 |
| H | -1.5294428 | 0.1092955 | -1.1345306 |
| C | 0.4929266 | -1.0751800 | -3.5946551 |
| C | -0.8248250 | -1.8102274 | 3.6423062 |
| H | -0.3564092 | -2.7857210 | 3.8511267 |
| H | -1.0669540 | -1.3921364 | 4.6350013 |
| C | -2.1113064 | -2.0378723 | 2.8511411 |
| H | -1.8995334 | -2.4683923 | 1.8602563 |

| | | | |
|---|---|---|---|
| H | -2.7676775 | -2.7371967 | 3.3871692 |
| H | -2.6724273 | -1.1048185 | 2.6970675 |
| C | 3.8583354 | 0.0370094 | 3.6426252 |
| H | 3.7964027 | 0.6772385 | 4.5395320 |
| H | 4.0799521 | -0.9700649 | 4.0293588 |
| C | 5.0025230 | 0.5119168 | 2.7464652 |
| H | 4.8814878 | 1.5605435 | 2.4384803 |
| H | 5.9612651 | 0.4336983 | 3.2781240 |
| H | 5.0705283 | -0.0978840 | 1.8325979 |
| N | 1.7263214 | -1.4304971 | 4.8331310 |
| H | 1.6882546 | -2.4560182 | 4.8633091 |
| C | 1.4146162 | -0.7616793 | 6.0015370 |
| O | 1.1381673 | 0.4438124 | 6.0371840 |
| N | 1.4596253 | -1.5751670 | 7.1310428 |
| H | 1.6006714 | -2.5718092 | 6.9170468 |
| C | 0.6660742 | -1.3226344 | 8.2745860 |
| C | -0.2265007 | -2.3174063 | 8.7179541 |
| C | 0.7070008 | -0.1317294 | 9.0024916 |
| C | -1.0606491 | -2.1459591 | 9.8628962 |
| C | -0.1239660 | 0.0588303 | 10.1298562 |
| H | 1.3847043 | 0.6565775 | 8.6792143 |
| C | -1.0035159 | -0.9276408 | 10.5709238 |
| C | -1.8166618 | -3.3598319 | 9.9994443 |
| C | -1.4256166 | -4.1976796 | 8.9756576 |
| H | -0.0640286 | 1.0068237 | 10.6677251 |
| H | -1.6345073 | -0.7624908 | 11.4460753 |
| H | -2.5458874 | -3.5923594 | 10.7697177 |
| H | -1.7252563 | -5.2168041 | 8.7532413 |
| N | -0.4907133 | -3.5621896 | 8.1862211 |
| H | 0.0754948 | -4.0055448 | 7.4583155 |
| C | -1.9785455 | -1.5303767 | -3.2985317 |
| H | -2.4881365 | -0.8599016 | -4.0113824 |
| H | -1.7215275 | -2.4263141 | -3.8839889 |
| C | -2.9343549 | -1.9090146 | -2.1622402 |
| H | -2.4258121 | -2.5301944 | -1.4088280 |
| H | -3.3379322 | -1.0268628 | -1.6458145 |
| H | -3.7876747 | -2.4804535 | -2.5536240 |
| C | 2.9701883 | -0.8099899 | -3.9944104 |
| H | 3.0589095 | -1.8999425 | -4.1332596 |
| H | 2.8182316 | -0.4071063 | -5.0114895 |
| C | 4.2837043 | -0.2846880 | -3.4199545 |
| H | 4.2932229 | 0.8123271 | -3.3425966 |
| H | 4.4684531 | -0.6966179 | -2.4164224 |
| H | 5.1231727 | -0.5842011 | -4.0624344 |
| N | 0.4641354 | -1.8336639 | -4.8019963 |
| C | -0.0013424 | -1.2559408 | -5.9696335 |
| O | -0.6287352 | -0.1920290 | -6.0004196 |
| N | 0.3000598 | -2.0067798 | -7.1071312 |
| H | 0.9242832 | -2.8024609 | -6.9188225 |
| H | 1.1927587 | -2.5477314 | -4.9200052 |
| C | 0.5240876 | -1.3751751 | -8.3571089 |
| C | 1.7973165 | -1.4653294 | -8.9490458 |
| C | -0.4496643 | -0.6456524 | -9.0395096 |
| C | 2.1060489 | -0.8520043 | -10.2000419 |
| C | -0.1560839 | -0.0196356 | -10.2719959 |
| H | -1.4413263 | -0.5557285 | -8.5983863 |
| C | 1.1031097 | -0.1136593 | -10.8607390 |
| C | 3.4809433 | -1.1629432 | -10.4763000 |
| C | 3.9405623 | -1.9309108 | -9.4264323 |
| H | -0.9437282 | 0.5477883 | -10.7712324 |
| H | 1.3104382 | 0.3751703 | -11.8144116 |
| H | 4.0536110 | -0.8726491 | -11.3521267 |
| N | 2.9385362 | -2.0898602 | -8.4925192 |
| H | 2.9623690 | -2.7280672 | -7.6932138 |
| H | 4.9070212 | -2.4006554 | -9.2739020 |

| | | | |
|---|---|---|---|
| C | 3.8178938 | -8.7818804 | 3.4908956 |
| C | 4.8699246 | -7.9669496 | 3.0170585 |
| C | 4.6193287 | -7.1446295 | 1.9136735 |
| C | 3.3655508 | -7.1146296 | 1.2954728 |
| C | 2.3406567 | -7.9158171 | 1.8049073 |
| C | 2.5362655 | -8.7630441 | 2.9016549 |
| H | 5.4090664 | -6.4975441 | 1.5314435 |
| H | 1.3621416 | -7.8759948 | 1.3259081 |
| C | 3.1362205 | -6.2723581 | 0.0607801 |
| H | 3.7984953 | -5.3978195 | 0.0783672 |
| H | 2.1110404 | -5.8800582 | 0.0576919 |
| C | 3.3791286 | -7.0816083 | -1.1933518 |
| C | 4.6682667 | -7.1985206 | -1.7205477 |
| C | 2.3267977 | -7.7597664 | -1.8158178 |
| C | 4.9377903 | -7.9904992 | -2.8425203 |
| H | 5.4804002 | -6.6516242 | -1.2408624 |
| C | 2.5401991 | -8.5640579 | -2.9398581 |
| H | 1.3177829 | -7.6381316 | -1.4206040 |
| C | 3.8583968 | -8.6790378 | -3.4293811 |
| C | 1.4222796 | -9.6246954 | 3.4532959 |
| H | 1.8381259 | -10.6200077 | 3.6796508 |
| H | 1.1278604 | -9.2091570 | 4.4310434 |
| C | 0.1859885 | -9.7687300 | 2.5648621 |
| H | 0.4439386 | -10.1635658 | 1.5710085 |
| H | -0.5309914 | -10.4602915 | 3.0281446 |
| H | -0.3318318 | -8.8087823 | 2.4230778 |
| C | 6.2141084 | -7.9964628 | 3.7183131 |
| H | 6.0526616 | -7.7596444 | 4.7843626 |
| H | 6.5976154 | -9.0315478 | 3.7135584 |
| C | 7.2930513 | -7.0763346 | 3.1481018 |
| H | 6.9915237 | -6.0201499 | 3.1849060 |
| H | 8.2182978 | -7.1773679 | 3.7308480 |
| H | 7.5261777 | -7.3252866 | 2.1026953 |
| N | 4.0863230 | -9.6482133 | 4.5972300 |
| H | 4.8950260 | -10.2608085 | 4.5202232 |
| C | 3.6007321 | -9.3922290 | 5.8493112 |
| O | 2.7735376 | -8.4745577 | 6.0527627 |
| N | 4.0472675 | -10.2059629 | 6.8555050 |
| H | 4.6741837 | -10.9755374 | 6.6323317 |
| C | 3.8401626 | -9.8691740 | 8.2245530 |
| C | 3.1219855 | -10.7287263 | 9.0672189 |
| C | 4.2989795 | -8.6670675 | 8.7620059 |
| C | 2.8670801 | -10.4044860 | 10.4352906 |
| C | 4.0311825 | -8.3189736 | 10.1010815 |
| H | 4.8511283 | -7.9807115 | 8.1187062 |
| C | 3.3245467 | -9.1738625 | 10.9415107 |
| C | 2.0846322 | -11.4826009 | 10.9707234 |
| C | 1.8918904 | -12.3903606 | 9.9509662 |
| H | 4.3676853 | -7.3499516 | 10.4700891 |
| H | 3.1024539 | -8.8834326 | 11.9686587 |
| H | 1.7000273 | -11.5698399 | 11.9821259 |
| H | 1.3549934 | -13.3339197 | 9.9417229 |
| N | 2.5175055 | -11.9361273 | 8.8040506 |
| H | 2.5153628 | -12.4113615 | 7.9084026 |
| C | 1.4123776 | -9.2838784 | -3.6517643 |
| H | 1.3829054 | -8.9259667 | -4.6959918 |
| H | 1.6623533 | -10.3556546 | -3.7264761 |
| C | 0.0251321 | -9.1485803 | -3.0253862 |
| H | 0.0070738 | -9.5399027 | -1.9978310 |
| H | -0.3108411 | -8.1032564 | -2.9934831 |
| H | -0.7098750 | -9.7147336 | -3.6130952 |
| C | 6.3239229 | -8.1076036 | -3.4379332 |
| H | 6.5280787 | -9.1741256 | -3.6269203 |
| H | 6.3009255 | -7.6397695 | -4.4370401 |
| C | 7.4605604 | -7.5007218 | -2.6156510 |

| | | | |
|---|---|---|---|
| H | 7.3452909 | -6.4138883 | -2.4953205 |
| H | 7.5176055 | -7.9477494 | -1.6121003 |
| H | 8.4222036 | -7.6740115 | -3.1176711 |
| N | 4.0903426 | -9.5290532 | -4.5582280 |
| C | 4.2305183 | -9.0273334 | -5.8202678 |
| O | 4.3216655 | -7.7946077 | -6.0305354 |
| N | 4.2933777 | -9.9456624 | -6.8345844 |
| H | 4.2516471 | -10.9351432 | -6.6005359 |
| H | 3.8314290 | -10.5083815 | -4.4628690 |
| C | 4.0368476 | -9.5697783 | -8.1873821 |
| C | 5.0246017 | -9.7294977 | -9.1674492 |
| C | 2.8164316 | -9.0195939 | -8.5775594 |
| C | 4.8022371 | -9.3604002 | -10.5299843 |
| C | 2.5903826 | -8.6242792 | -9.9111247 |
| H | 2.0384402 | -8.8796029 | -7.8258364 |
| C | 3.5660219 | -8.7931489 | -10.8887279 |
| C | 6.0264252 | -9.6288297 | -11.2299707 |
| C | 6.9212197 | -10.1262788 | -10.3064981 |
| H | 1.6410218 | -8.1537962 | -10.1679136 |
| H | 3.3877553 | -8.4612929 | -11.9120145 |
| H | 6.2268849 | -9.4572113 | -12.2829692 |
| N | 6.3170350 | -10.1875758 | -9.0637840 |
| H | 6.7605315 | -10.5067367 | -8.2096986 |
| H | 7.9519654 | -10.4447608 | -10.4275467 |

## UV–Vis and Fluorescence Studies

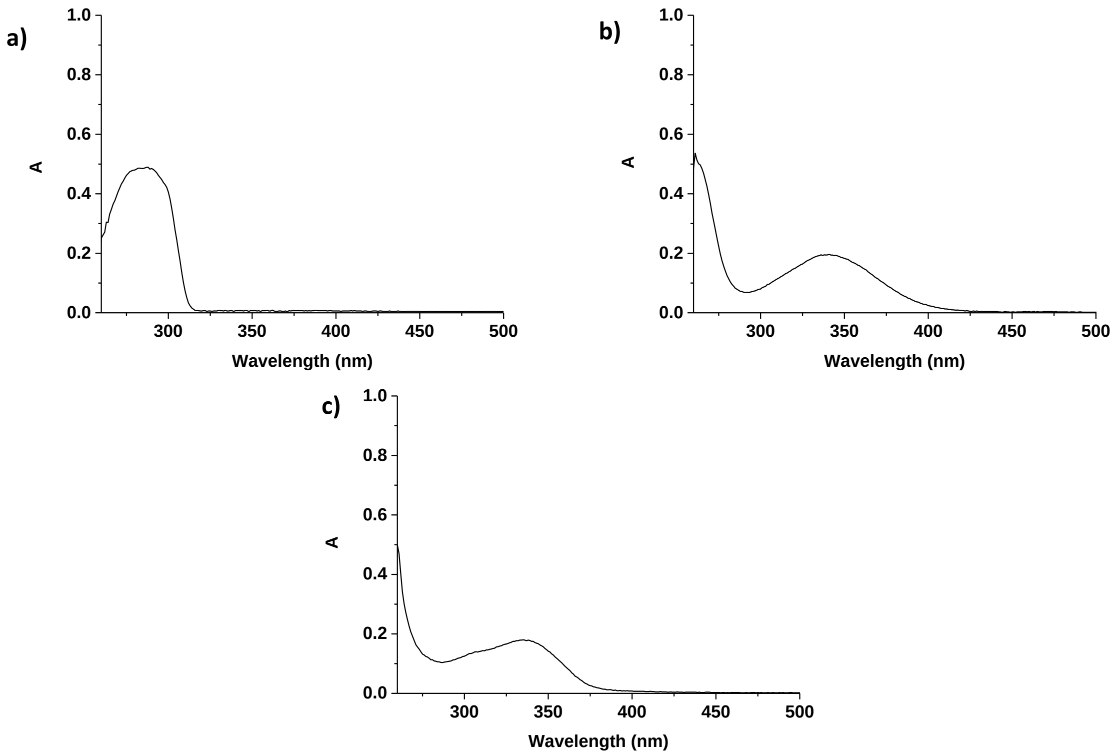


**Figure S14.** UV–Vis spectra of (**a**) **L1**, (**b**) **L2**, and (**c**) **L3** in DMSO (M = 2.5 ·$10^{-5}$ M).

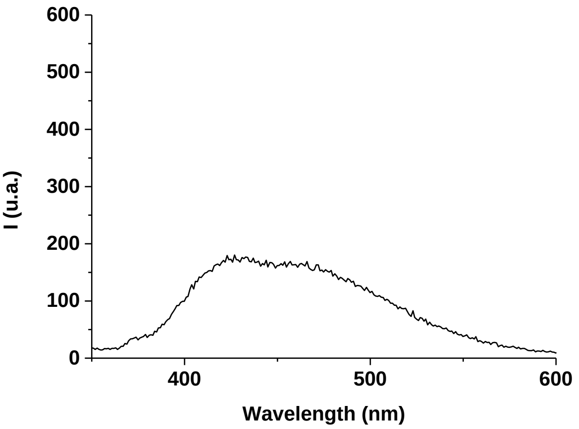


**Figure S15.** Fluorescence spectrum of **L3** (M = 2.5 ·$10^{-5}$ M) in DMSO ($\lambda_{exc}$ = 340 nm).

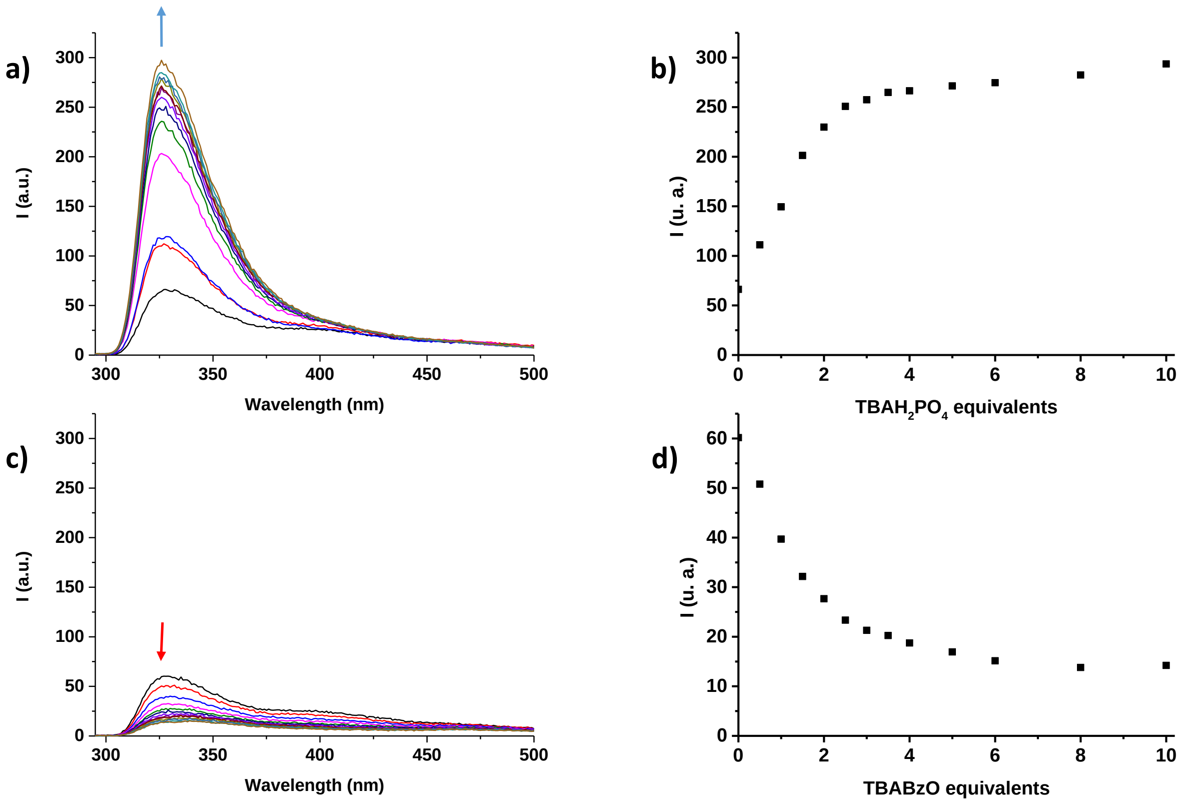


**Figure S16**. Fluorescence titration of **L1** (2.5 ·$10^{-5}$ M) in DMSO in the presence of (**a**) $TBAH_2PO_4$ and (**b**) TBABzO ($\lambda_{exc}$ = 278 nm). Plot of the fluorescence emission maximum (328 nm) as a function of (**c**) $TBAH_2PO_4$ and (**d**) TBABzO equivalents added.
